# Temporal response characterization across individual multiomics profiles of prediabetic and diabetic subjects

**DOI:** 10.1038/s41598-022-16326-9

**Published:** 2022-07-15

**Authors:** Minzhang Zheng, Carlo Piermarocchi, George I. Mias

**Affiliations:** 1 Biochemistry and Molecular Biology, Michigan State University, East Lansing, MI 48824 USA; 2 Institute for Quantitative Health Science and Engineering, Michigan State University, East Lansing, MI 48824 USA; 3 Physics and Astronomy, Michigan State University, East Lansing, MI 48824 USA

**Keywords:** Molecular medicine, Diagnostic markers, Personalized medicine, High-throughput screening

## Abstract

Longitudinal deep multiomics profiling, which combines biomolecular, physiological, environmental and clinical measures data, shows great promise for precision health. However, integrating and understanding the complexity of such data remains a big challenge. Here we utilize an individual-focused bottom-up approach aimed at first assessing single individuals’ multiomics time series, and using the individual-level responses to assess multi-individual grouping based directly on similarity of their longitudinal deep multiomics profiles. We used this individual-focused approach to analyze profiles from a study profiling longitudinal responses in type 2 diabetes mellitus. After generating periodograms for individual subject omics signals, we constructed within-person omics networks and analyzed personal-level immune changes. The results identified both individual-level responses to immune perturbation, and the clusters of individuals that have similar behaviors in immune response and which were associated to measures of their diabetic status.

## Introduction

The development of novel technologies in personal health monitoring devices, high throughput sequencing and computational methods has generated massive omics data, and provides both a great opportunity and challenge to precision health^1–5^. The big data provides plentiful health information ranging from biomolecular, physiological, and environment data to clinical measures. This information helps identify potential deviations from a healthy baseline and improves health risk predictions^1^. A big challenge of a big data approach to precision health is how to integrate and understand these multi-dimensional, extremely diverse sources, with highly heterogeneous data^3^. Early efforts by Chen, Mias, Li-Pook-Than et al. focused on assessing the feasibility of integrated Personal Omics Profiling (iPOP), by utilizing a multiomics integration framework to interpret healthy and diseased states followed through an individual’s blood-based multiomics assessment^6^. More recent efforts by Sara Ahadi et al. revealed personal aging markers by using deep longitudinal profiling^7^, Abdellah Tebani et al. discovered how the personal cohort changes during the wellness period^8^. Environmental effects have also been studied by M. Reza Sailani et al., revealing two biological seasonal patterns in California by multiomics profiling^9^. Wearable sensors have also been used in digitalized health in tracking physiomes and activity^10^. Other implementations have used multiomics to monitor the drug responses^11^. Non-invasive longitudinal saliva multiomics have been recently used by Mias et al. to monitor immune responses in a vaccinated individual^12^. Although these efforts have shown the great promise of deep multiomics profiling, the complexity of data presents limitations for practical implementations. Deep multiomics data come from diverse sources, and have different types, sizes and ranges, which complicates comparisons between different individuals’ personal multiomics. In the Pioneer study by Price et al.^13^ dynamic data clouds were used for longitudinal monitoring of individual subjects, in a study that also incorporated behavioral coaching to improve clinical biomarkers. In recent work, Zhou et al.^14^ carried out iPOP across multiple individuals, and built correlation networks of molecular associations. However, to the best of our knowledge, direct networks of individuals associated with longitudinal individual deep multiomics profiles have not been constructed.

In this investigation we took an individual-focused approach to categorize personal longitudinal deep multiomics profiles and group individuals into communities, using spectral representations of individual multiomics time series. In taking this individual-focused approach, one of our goals was to perform an analysis closer to clinical applications, where inherently the individual is monitored over time to enable a personal diagnosis. We implemented this approach on personal multiomics profiling data from prediabetic or diabetic individuals (type 2 diabetes mellitus, T2D) at its earliest stage from the study by Zhou et al.^14^. We first identified individual-level molecular responses to immune perturbation associated to individual physiological state changes. Based on the individual temporal responses, we built clusters across individuals showing similar trends. The microscopic molecular behavior was linked to phenotypic differences, including body mass index and insulin resistance, with the immune response dominating differences attributed to diabetic status.

## Methods

### Summary of cohort details and data

The original data used in this analysis comes from the study by Zhou et al.^14^, that focused on multiomics characterization of host-microbe dynamics in prediabetics. The measures, SSPG, Matsuda, DI and isrMax, came from the other paper of the same project by Shussler-Fiorenza Rose et al.^4^. All data obtained were made publicly available by the original authors^4,14^ as described therein (under Stanford IRB No. 23602), and no additional institutional review board approvals (IRB) were required for this investigation.

**Figure 1:**
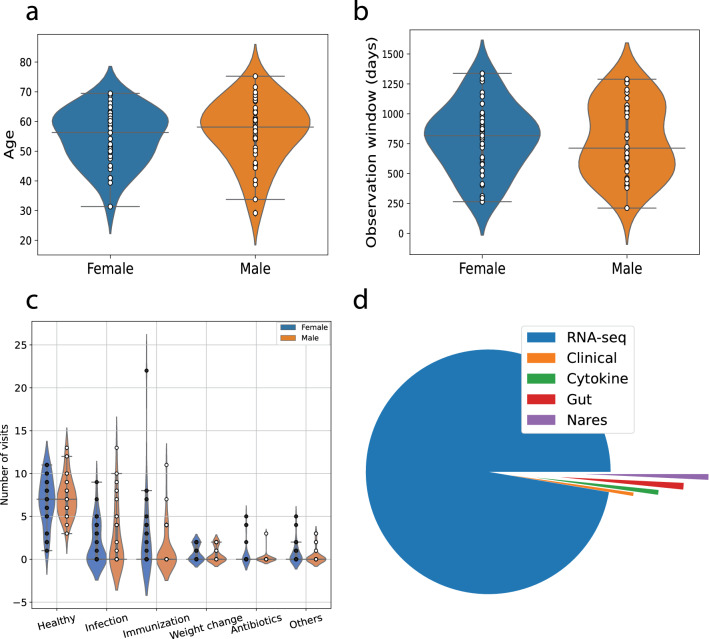
Cohort description. Summary distributions across sexes for (**a**) Age, (**b**) observation window, and (**c**) visits for different conditions. (**d**) Proportion of time series from different data modalities.

The participants had been classified as diabetic or prediabetic in the original study according to one of the following three criteria^14^: (i) haemoglobin A1C (A1C; diabetic $\ge 6.5\%>$ prediabetic $\ge 5.7\%$), (ii) fasting glucose (diabetic $\ge$ 126 mg $\hbox {dl}^{-1}>$ prediabetic $\ge 100$ mg dl $^{-1}$) and (iii) based on an annual oral glucose tolerance test (OGTT; diabetic $\ge$ 200 mg $\hbox {dl}^{-1}>$ prediabetic $\ge 140$ mg $\hbox {dl}^{-1}$ at 2 h). The different subjects in the study had highly heterogeneous visit records: some subjects only had one visit record but one subject has more than 150 visit records (time points), which is about 15 times more than the average of 10 visits per subject. Multiple omics were generated from the subjects including blood based transcriptomics, microbiome data (nares/gut), cytokine measurements and clinical measures. To ensure the individual omics profiles had enough time points and time series, we filtered records from individuals so that the number of time points $N_t >= 4$ and the number of omics $N_o > 500$, across participants from the data source^14^. We also excluded the subject with the 150+ visits records, as this was not comparable directly to other subjects in our analysis, given the density of points. Our final filtered dataset contained 69 subjects from the original data source. Figure 1a shows the age distribution across sexes for the subjects. We had 34 males and 35 females, and most subjects were older than 50. Summary distributions of the subjects’ observation window are shown in Fig. 1b. The observation window is heterogeneous, ranging from 200 to 1200 days. During the observation window, there were a total of 846 visits with different conditions, including: 486 healthy visits as baseline, 148 visits when subjects got infected, 119 visits had immunization effects, 43 visits with subject weight gain or loss period, 18 visits with subjects on antibiotics, and 32 other healthy conditions, summarized in Fig. 1c. Overall we analyzed 733,425 time series across 5 datasets: 713,874 RNA-sequencing (RNA-seq) data, 3221 clinical measures, 4554 cytokines, 6336 Gut and 5440 Nares measurements. The majority ($>90$%) of the time series comes from RNA-seq, Fig. 1d. As RNA-seq provides a comprehensive and accessible map of the transcriptome, with more omics profiled (by number comparison) compared to other-omes (e.g. proteomes/metabolomes, etc.) we expect that the majority of future omics profiling studies data will follow similar trends (as has been the case so far), though we do expect more microbiome data to emerge, as the host-microbiome interaction is still under considerable investigation.

### Data preprocessing

**Figure 2:**
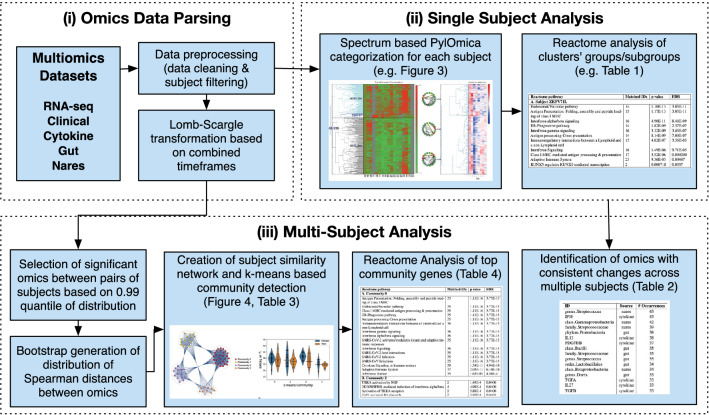
Workflow. Following the initial parsing of multiple omics datasets (i), our workflow has two main branches: (ii) single subject analysis and (iii) multi-subject similarity analysis, with examples of the output shown and relevant figures and tables.

To obtain an individual’s omics profile, we combined all the omics source dataset into a dataframe, then separated into dataframes for each individual. Since our workflow has two branches: single subject analysis and multi-subject similarity analysis, as seen in Fig. 2, the following data preprocessing was carried out for the two branches: (i) For the single subject analysis, we selected the signals with less than 25% time points missing from each individual’s dataframe as the input for single subject analysis, using each individual’s time points as possible measurement points. (ii) For multi-subject analysis, we sorted each individual’s time frame from their dataframes, then combined all individual time frames to get all the possible time points as the common time frame. We then calculated Lomb–Scargle periodograms from each individual’s dataframe using this common time frame as the set of possible measurement points. The transformed dataframe was then used as the input for the multi-subject analysis.

### Individual subject analysis

#### Time series categorization

Individual subject analysis was carried out in Python, with the package PyIOmica utilities for time series categorization^15^ (command calculateTimeSeriesCategorization). Briefly, for each subject *s*, for each omics *i* its time series *X* was analyzed over its constituent timepoints. The individual omics intensities at timepoint *j* were compared to the initial timepoint by subtracting its intensity from all values, $\tilde{X}_{is}(t_j)=X_{is}(t_j)-X_{is}(0)$. The data were then normalized to a time series *Q*, using the Euclidean norm, so that $Q_{is}(t_j)=\frac{\tilde{X}_{is}(t_j)}{\sqrt{\sum _k\left( \tilde{X}_{is}(t_k)^2\right) }}$.

The algorithm’s classification of signals into trends uses spectral methods, as previously described^12,15^. Briefly, for each signal a Lomb–Scargle periodogram is calculated as a list $P_{is}$. The inverse Fourier transform of $P_{is}$ returns a list of autocorrelations, $\{\rho _{isk}\}$, where $k\in \{0,\ldots , N/2\}$ is the lag. In parallel with the original time series signals, a bootstrap set of $10^5$ time series was generated by sampling from the original data with replacement. The autocorrelations at different lags of the bootstrap set were computed to generate an autocorrelation null distribution for each lag from which a set of cutoffs $\{\rho _{ck}\}$ were obtained corresponding to a 0.95 quantile. A time series was then assigned to a class labeled with the lowest *Lag* *l* for which the series’ autocorrelation $\rho _{isl}$ is larger than the cutoff, i.e., where $l=\text {Min}\left[ \left\{ j:\rho _{\text {isj}}\ge \rho _{\text {cj}}\right\} \right]$, and $j\in {1,\ldots ,k}$.

If a time series $\tilde{X_{is}}$ does not have autocorrelations that satisfy the cutoff criteria, the algorithm then checked if the series has a pronounced peak or trough at any time point. The time series’ maximum, $\max _{is}=\max \tilde{X_{is}}$, and minimum, $\min _{is}=\min \tilde{X_{is}}$ were compared to $\left\{ \text {max}_{cn},\text {min}_{cn}\right\}$, which are maxima and minima cutoffs from null distributions again computed using the bootstrap time series for all possible time series lengths *n*. $\tilde{X_{is}}$ is then labeled as a *SpikeMax* signal if $\max _{is}>\text {max}_{cn}$, or as a *SpikeMin* if $\min _{is}<\text {min}_{cn}$. A time series that did not meet any of the cutoff criteria was not labeled as having a statistically significant trend. The approach categorizes each omics signal based on its own trend, and is thus not affected by differences in the omics modality, making it directly extensible to incorporating different kinds of time series data^16^.

#### Clustering and heatmaps

**Figure 3:**
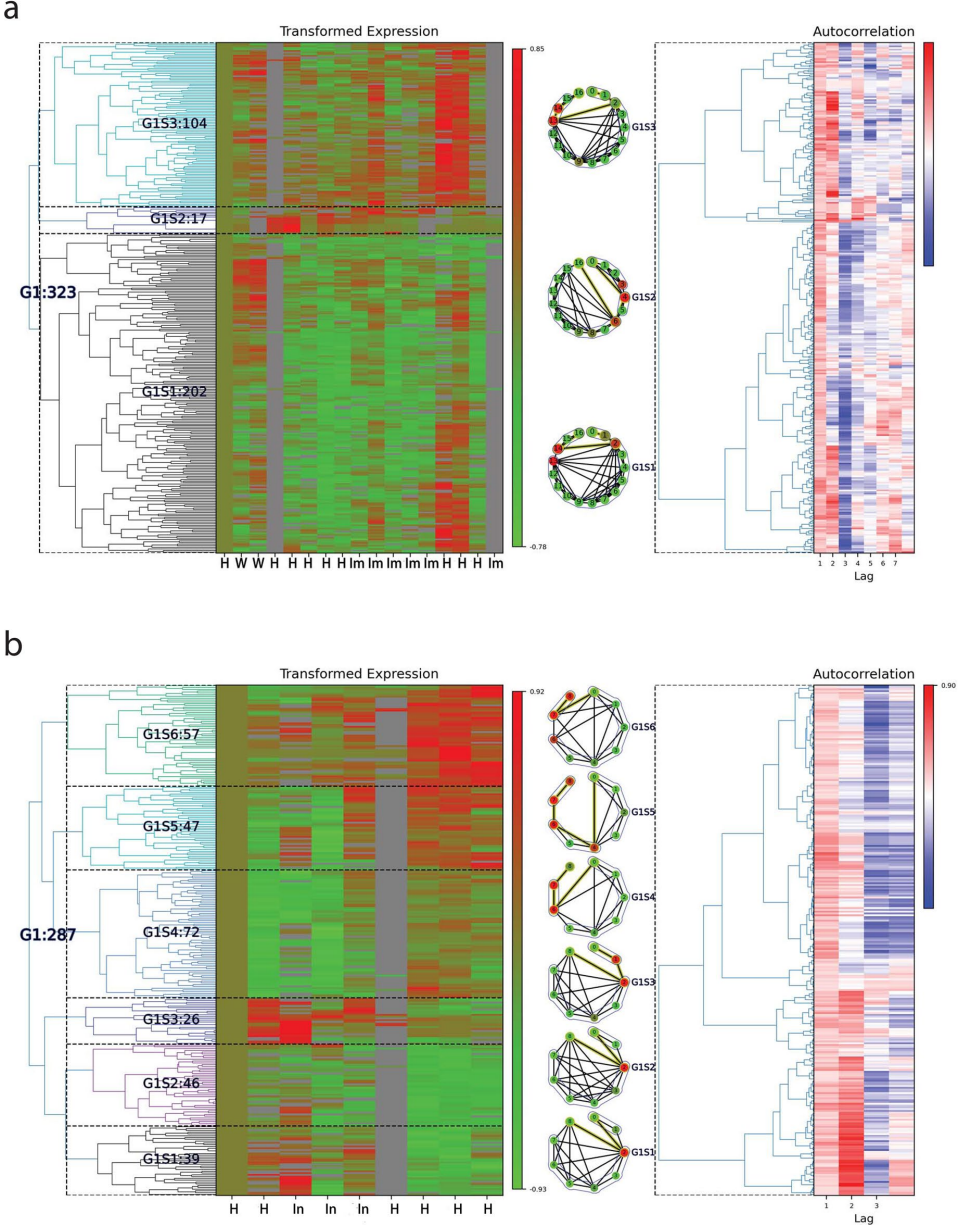
Single individuals’ multiomics clusters. Two examples of Lag 1 classification outcomes are shown for (**a**) Subject ZKFV71L and (**b**) Subject ZTMFN3O. In these examples the information is summarized as follows: *Left panel*: the cluster of groups/subgroups for Lag 1 class are shown in the visits time frame. The visit time points have been labeled by healthy status, where H: Healthy, W: Weight gain/loss, Im: Immunization, In: Infection. *Middle panel*: the community structure of visits within each subgroup, where the community structure is based on our visibility-graph-based community detection algorithm^24^. *Right panel*: corresponding autocorrelations for the time series shown.

After classification, and using PyIOmica’s clusterTimeSeriesCategorization function, we carried out a two-tier hierarchical clustering (agglomerative; complete linkage) for each temporal class, to identify groups (G) and sub-groups (S). The clustering grouping used a similarity based on $\{\rho _{isk}\}$ (for the autocorrelation classification) and $\{Q_{is}\}$ for the second tier. Groups and subgroups were determined using a silhouette algorithm^17^. The results were visualized for each subject and every temporal class identified using PyIOmica’s visualizeTimeSeriesCategorization. Example outputs are shown in Fig. 3 and included in the Online Data Files (ODFs). The first tier of clustering aims at capturing the autocorrelation structure, and hence the dominant pattern in the data. The second tier clustering based on the data values can distinguish pattern variations, and particularly sign differences that the autocorrelations (being the inverse Fourier transform of the periodogram frequencies) would not capture^16^.

#### Reactome enrichment analysis

Reactome^18^ pathway enrichment analysis was carried out for each Group/Subgroup and each subject using the Reactome application programming interface (API) in PyIOmica. Examples are shown in Table 1 for two subjects, and complete output for all subjects is included in the ODFs.

### Across subject comparisons

#### Individual results aggregation

The individual subject omics that showed statistically significant trends were aggregated to identify consistency across individuals. Signals identified as having statistically significant trends, $FDR < 0.05$, in more than 50% of the individuals, are shown in Table 2.

#### Network construction

The network analysis was carried out in Python, using networkx^19^ and scikit-network^20^. First the time series periodograms for all the omics time series were computed for all subjects using the LombScargle function in PyIOmica. Next, for pairs of subjects *p*, *q* and for each omics time series *i*, a Spearman correlation matrix $S_i$ was constructed. In parallel, a bootstrap simulation of 50,000 time series was also generated from the data, as a null distribution, and the pairwise Spearman correlations were computed for these as well to determine a Spearman correlation cutoff for significance, $s_c$ at the 0.99 quantile level. Entries were kept that were most correlated to each other, by creating a restricted distance matrix $R_{i}$, such that

1 $$\begin{aligned}{}[R_{i}]_{p,q} = {\left\{ \begin{array}{ll} 1, \text {if } [S_i]_{p,q} > s_c\\ 0, \text {if } [S_i]_{p,q} \le s_c~. \end{array}\right. } \end{aligned}$$

A weighted network was then constructed, with the subjects represented as nodes, with an adjacency matrix *A*, constructed as $A = \sum _i R_i$. The entries *p*, *q* of the adjacency matrix represent the connections in the network. A nonzero entry $A_{p,q}$ means there is an edge connecting nodes (subject) *p* and *q*. The magnitude of $A_{p,q}$ provides a weight of the edge. In summary, the edges connecting pairs of nodes (subjects) were added to the network if there was at least one omics for which the Spearman correlation between the subjects was greater than $s_c$, and the edges were weighted by the number of omics that met this criterion for each pair of subjects. Thus, the network captures the similarity between subjects, putting more weight on edges connecting subjects with a higher number of similar omics signals.

#### Network communities calculation

To determine the network community structure, a k-means approach was used. The computation used scikit-network’s clustering.kmeans, applying an embedding, and utilizing singular value decomposition with dimension one. The number of communities (4) was selected based on the elbow and silhouette methods^17^, and the sklearn.metrics.silhouette_score^21^ was used for the silhouette scores calculation.

#### Mann-Whitney U tests

Subject measurements were compared between members of the communities in the network calculations (see above). We used non-parametric Mann–Whitney U tests^22^, to test for statistically significant pairwise differences across communities (*p* value < 0.05). The results shown in Table 3 were computed using the scipy.stats.mannwhitneyu Python functionality^23^.

## Results

### Single subject analysis

We used PyIOmica’s^15^ spectral methods to classify the time series for each individual’s omics into temporal trends. The objective is to identify sets of omics that show similar temporal behavior that deviates from each individual’s own baseline. The PyIOmica categorization algorithm generates 3 sets of classes from an individual’s omics time series: (i) *Lag classes*, of time series showing statistically significant autocorrelation at different lags, (ii) *Spike Maxima class* of time series with no autocorrelation but with positive spikes (high intensity pulses), and (iii) *Spike Minima class* of time series with no autocorrelation but with negative spikes (low intensity pulses)^15,16^. Then within each class, the algorithm separates time series into groups and subgroups based on the autocorrelation patterns and signal intensities. Further details are provided in the “Methods” section.

All analyses and results from each individual’s classifications are provided in the Online Data Files (ODFs). Here we show examples of Lag1 classification for two subjects, ZKFV71L (Female, 66-year old, Prediabetic) and ZTMFN3O (Female, 40-year old, Prediabetic), Fig. 3a and b respectively. The Lag 1 class for subject ZKFV71L has 323 time series, which were assigned to 1 group and 3 subgroups, shown in Fig. 3a left panel. The intensity changes of the 3 subgroups represented the healthy status changes, indicating the systemic immune responses in the subject. Within each subgroup, we created a mean time series, whose intensities of each time point equals to the average intensity of the time series at this time point within the subgroups. These are used to obtain the time points community structure using our visibility-based community detection algorithm^24^. The communities of each subgroup were related to the health status changes, as indicated in Fig. 3 middle panel (circular visibility graph layouts are shown for each subgroup’s mean time series). The autocorrelation heatmap corresponding to the time series in this category is also shown in Fig. 3 right panel. Similar results were found in subject ZTMFN30, Fig. 3b. The results for all other subjects, including corresponding omics in each class and group/subgroup classifications, and visualizations are all available in the ODFs.

Once sets of omics that show similar profiles in time are identified, we can assess the biological significance of these temporal associations. Following classification, we carried out Reactome pathway^18^ enrichment analysis for the genes that showed statistically significant trends for each subject. The statistically significant (False Discovery Rate, *FDR* <  0.05) pathways results for subject ZKFV71L and ZTMFN3O for the autocorrelation Lag 1 class are shown in Table 1. The over-representation for subject ZKFV71L included Endosomal/Vacuolar pathway (14 genes), Antigen Presentation: Folding, assembly and peptide loading of class I MHC (15 genes), Interferon alpha/beta signaling (16 genes), ER-Phagosome pathway (14 genes), Interferon gamma signaling (16 genes), Antigen processing-Cross presentation (14 genes), Immunoregulatory interactions between a Lymphoid and a non-Lymphoid cell (15 genes), Interferon Signaling (16 genes), Class I MHC mediated antigen processing and presentation (17 genes) and Adaptive Immune System (23 genes), etc. These Reactome pathways indicated immune responses of this subject (ZKFV71L) corresponding to the health status change from Healthy to Immunization. Similarly, we also found statistically significant Reactome pathways for subject ZTMFN3O, including: Cellular response to heat stress (7 genes), Cellular responses to stress (12 genes), Cellular responses to stimuli (12 genes), Interleukin-10 signaling (7 genes), Signaling by Interleukins (13 genes) and Cytokine Signaling in Immune system (14 genes). These pathways are also indicative of an immune response of this subject (ZTMFN3O) corresponding in this case to a health status change from Healthy to Infection. Reactome Pathway enrichment analyses for all subjects including all subgroups are included in the ODFs.

### Multi-subject similarity analysis

Based on the individual results, we first aggregated the omics that showed statistically significant trends in each individual to identify the signals that are consistent across the majority of individuals ($>50$% of subjects, number of occurrences $\ge 35$), included in Table 2. We found that high frequency signals came from 3 data sources: cytokines, nares and gut microbiome. Many studies have now shown that cytokines have a profound relationship with type 2 diabetes^25–27^, our findings are consistent with these previous works and provide potential biomakers for type 2 diabetes (see also Discussion).

**Figure 4:**
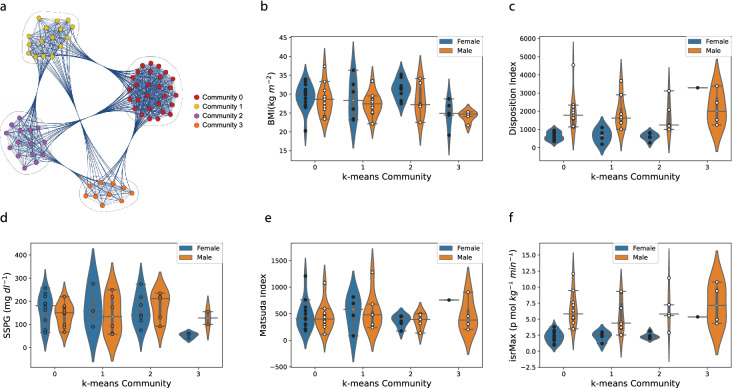
Similarity analysis across individuals. (**a**) The k-means based community structure of the subjects’ similarity network (nodes represent subjects and weighted edges omics showing similar temporal behavior across individuals). (**b**)–(**f**) Distributions of five types of measures in the 4 network communities by gender: (**b**) BMI; (**c**) DI, disposition index; (**d**) SSPG, steady-state plasma glucose; (**e**) Matsuda index and (**f**) isrMax, maximum insulin secretion rate.

To further investigate common responses across individuals, we created a multi-subject similarity network. The network was constructed by comparing the Spearman correlation of the spectral time series representation (periodogram) across common omics between every pair of subjects. The network, shown in Fig. 4a, has nodes corresponding to the 69 subjects, with weighted edges corresponding to the number of omics that showed similar temporal behavior. We used a k-means algorithm to calculate communities of the network (see “Methods” section for details). Four communities were identified, denoted as Community 0, Community 1, Community 2 and Community 3. Community 0 has 28 individuals, 15 Females and 13 Males, ages from 29 to 69, with disease status including 19 Prediabetic, 1 Diabetic, 5 Crossover and 3 Control. Community 1 has 16 individuals, 6 Females and 10 Males, ages from 33 to 75, 12 Prediabetic, 1 Crossover and 3 Control. Community 2 has 15 individuals, 9 Females and 6 Males, age from 39 to 67, 13 Prediabetic, 2 Crossover. Community 3 has 10 individuals, 5 Females and 5 Males, age from 39 to 70, 6 Prediabetic, 2 Crossover and 2 Control. We then compared clinical measures between the subjects in the community, including body mass index (BMI), disposition index (DI), steady-state plasma glucose (SSPG), Matsuda index (Matsuda), and maximum insulin secretion rate (isrMax). The violin plots show group separated by community and sex, Fig. 4b–f. These 5 distribution figures qualitatively indicate that the Community 0, Community 1 and Community 2 have similar distribution but have large differences with Community 3. We also found that the female and male subjects have different distributions, even within the same community for BMI, DI and isrMax measures. We carried out non-parametric Mann–Whitney U tests^22,28^ to compare across the different communities for statistical significance (*p* value < 0.05), with the results summarized in Table 3. The 5 measures do not show statistical significant differences between Community 0 and Community 1,Community 0 and Community 2, and Community 1 and Community 2. The BMI and SSPG distributions between Community 0 and Community 3, and between Community 2 and Community 3 have statistically significant differences, the BMI distributions between Community 1 and Community 3 also have statistically significant differences, indicating that the subjects in Community 3 have statistical difference in physiological states comparing with Community 0, Community 1 and Community 2. Differences in Male vs. Females in the comparisons, particularly for BMI, DI and isrMax, suggest that even though overall subjects in these three communities may have similar physiological states and responses, females and males still display different physiological states (though the low number of subjects is affecting power in further breaking down of community differences).

We next ranked the omics in each community (represented by the weighted edges), by their frequencies of occurrence. We then carried out Reactome Pathway Enrichment analysis for the top 25% ranked genes of each community (to reduce noise effects from low frequency genes). The statistically significant pathway results (FDR < 0.05) are shown in Table 4. Community 0 has 16 statistical significant Reactome pathways, related to immune response. For Community 2 the statistically significant Reactome pathways include TRKA activation by NGF, DDX58/IFIH1-mediated induction of interferon-alpha/beta, Activation of TRKA receptors, and Ca2+ activated K+ channels. Community 1 and Community 3 did not have statistically significant Reactome results. The Reactome pathway Enrichment analysis, along with the subjects’ BMI, DI, SSPG, Matsuda and isrMax distributions differences indicate that these affect temporal immune responses in different detected Communities, and also have sex differences (BMI and SSPG) between two of the identified communities (Community 0 and Community 3).

### Result summary

In summary, our results separate individuals with different physiological conditions and immune response based on the multiomics time series similarity of individual profiling analyses. We can detect changes in each individual compared to their own baseline that coincide with immune responses in each subject, and correspond to the individual time of onset per individual. The groups of genes discovered are associated with immune-relevant pathways, including antigen processing and presentation, interferon signaling and interleukin signaling (e.g., Table 1). The trends detected are consistently detected across multiple individuals (Table 2), and include multiple cytokines (IP10, IL13, PDGFBB, TGFA, IL27, TGFB, MCP1, GCSF, MIG, IL22, CD40L, VEGF, SCF and EGF) as well as bacterial signals (with top results by occurrence in the highest number of individuals including genus *Streptococcus* in both nares and gut and *Blautia* in gut, classes *Gammaproteobacteria* and *Betaproteobacteria* in nares, family *Streptococcaceae* in both nares and gut, and phyla *Proteobacteria* in gut and *Bacteroidetes* in nares, and order *Lactobacillales* in gut). Finally, a network with nodes representing subjects and weighted edges omics with similar temporal behavior across individuals, shown in Fig. 4, separated the individuals in 4 communities, with statistically significant differences (*p* value < 0.05) detected in BMI, SSPG, DI and isrMax, including sex differences, Table 3. The network communities detected involve different pathways for the top genes (highest weight edges) with similar behavior between subjects, Table 4. This involves in one group of subjects (Community 0) several immune responses (antigen processing and presentation, interferon and cytokine signaling, and several pathways also identified in SARS-CoV2-2 immune responses, as multiple immune-related genes are involved), and different pathways in a second group (Community 2, including TRKA activation by NGF, DDX58/IFIH1-mediated induction of interferon-alpha/beta, Activation of TRKA receptors and Ca2+ activated K+ channels).

**Table 1:** Statistically significant (*FDR* < 0.05) Reactome pathways results for subject ZKFV71L and ZTMFN3O for autocorrelation Lag 1.

Reactome pathway	Matched IDs	*p* value	FDR
**A. Subject ZKFV71L**			
Endosomal/Vacuolar pathway	14	1.10E−13	3.05E−11
Antigen Presentation: Folding, assembly and peptide loading of class I MHC	15	1.17E−13	3.05E−11
Interferon alpha/beta signaling	16	4.90E−11	8.48E−09
ER-Phagosome pathway	14	1.82E−09	2.37E−07
Interferon gamma signaling	16	3.32E−09	3.45E−07
Antigen processing-Cross presentation	14	8.14E−09	7.00E−07
Immunoregulatory interactions between a Lymphoid and a non-Lymphoid cell	15	4.82E−07	3.56E−05
Interferon Signaling	16	1.49E−06	9.71E−05
Class I MHC mediated antigen processing and presentation	17	3.52E−06	0.000200
Adaptive Immune System	23	9.36E−05	0.00487
RUNX3 regulates RUNX1-mediated transcription	2	0.000718	0.0337
**B. Subject ZTMFN3O**			
Attenuation phase	7	2.415E−10	5.121E−08
HSF1-dependent transactivation	7	1.152E−09	1.221E−07
Regulation of HSF1-mediated heat shock response	7	9.528E−08	6.670E−06
Cellular response to heat stress	7	3.135E−07	1.462E−05
HSF1 activation	5	3.482E−07	1.462E−05
Cellular responses to stress	12	4.469E−05	0.002
Cellular responses to stimuli	12	5.365E−05	0.002
RMTs methylate histone arginines	3	7.407E−04	0.019
Interleukin-10 signaling	7	2.416E−07	8.408E−05
Signaling by Interleukins	13	1.283E−05	0.0022
NR1H3 and NR1H2 regulate gene expression linked to cholesterol transport and efflux	4	0.00032	0.0353
Signaling by Nuclear Receptors	8	0.00057	0.0353
Cytokine Signaling in Immune system	14	0.00073	0.0353
Signaling by Overexpressed Wild-Type EGFR in Cancer	2	0.00073	0.0353
Inhibition of Signaling by Overexpressed EGFR	2	0.00073	0.0353
NR1H2 and NR1H3-mediated signaling	4	0.00083	0.0353
NR1H2 and NR1H3 regulate gene expression to limit cholesterol uptake	2	0.00114	0.0353
NR1H2 and NR1H3 regulate gene expression linked to gluconeogenesis	2	0.00114	0.0353
NR1H2 and NR1H3 regulate gene expression linked to triglyceride lipolysis in adipose	2	0.00114	0.0353
EGFR interacts with phospholipase C-gamma	2	0.00137	0.0357
Interleukin-18 signaling	2	0.00137	0.0357

**Table 2:** Frequency of signals with statistically significant temporal trends in individuals.

ID	Source	# Occurrences
genus_Streptococcus	Nares	43
IP10	Cytokine	43
class_Gammaproteobacteria	Nares	42
family_Streptococcaceae	Nares	39
phylum_Proteobacteria	Gut	38
IL13	Cytokine	38
PDGFBB	Cytokine	37
class_Bacilli	Gut	35
family_Streptococcaceae	Gut	35
genus_Streptococcus	Gut	35
order_Lactobacillales	Gut	34
class_Betaproteobacteria	Nares	34
genus_Dorea	Gut	33
TGFA	Cytokine	33
IL27	Cytokine	33
TGFB	Cytokine	33
phylum_Bacteroidetes	Nares	33
genus_Blautia	Gut	33
ALKP	Clinical	32
family_Micrococcaceae	Nares	32
MCP1	Cytokine	32
genus_Flavonifractor	Gut	32
class_Bacteroidia	Nares	32
order_Bacteroidales	Nares	32
family_Propionibacteriaceae	Nares	32
genus_Propionibacterium	Nares	32
genus_Clostridium.XlVa	Gut	32
GCSF	Cytokine	31
MIG	Cytokine	31
genus_Oscillibacter	Gut	31
IL22	Cytokine	31
genus_Clostridium.IV	Gut	31
CD40L	Cytokine	30
VEGF	Cytokine	30
order_Lactobacillales	Nares	30
SCF	Cytokine	30
EGF	Cytokine	30
family_Coriobacteriaceae	Gut	30
order_Coriobacteriales	Gut	30

**Table 3:** Mann–Whitney U test for different measures between two different communities.

Comparison	BMI	SSPG	Matsuda Index	Disposition Index	isrMax
C0 vs C1 Female	0.79	0.95	0.62	0.87	0.87
C0 vs C1 Male	0.60	0.91	0.76	0.90	0.27
C0 vs C1 Total	0.26	0.82	0.50	0.27	0.61
C0 vs C2 Female	0.09	0.90	0.70	0.97	0.77
C0 vs C2 Male	0.97	0.32	0.77	0.77	0.95
C0 vs C2 Total	0.19	0.57	0.53	0.97	0.91
C0 vs C3 Female	*0.015*	*0.0044*	0.43	0.14	0.14
C0 vs C3 Male	*0.049*	0.91	0.95	0.64	1.00
C0 vs C3 Total	*0.0017*	*0.012*	0.68	*0.013*	0.053
C1 vs C2 Female	0.39	0.83	0.26	1.00	0.91
C1 vs C2 Male	0.71	0.58	0.52	0.52	0.52
C1 vs C2 Total	0.086	0.67	0.17	0.23	0.56
C1 vs C3 Female	0.43	0.10	0.80	0.40	0.40
C1 vs C3 Male	*0.019*	1.00	0.68	0.93	0.15
C1 vs C3 Total	*0.033*	0.083	1.00	0.16	*0.037*
C2 vs C3 Female	*0.0040*	*0.017*	0.29	0.29	0.29
C2 vs C3 Male	0.052	0.57	1.00	0.29	1.00
C2 vs C3 Total	*0.00079*	*0.019*	0.51	*0.0087*	0.15

The labels C0, C1, C2,C3 correspond to Community 0, Community 1 Community 2 and Community 3, respectively. Statistically significant (*p* value < 0.05) results are shown in italics.

**Table 4:** Statistically significant (FDR < 0.05) Reactome pathways results of top quartile highest genes for Communities 0 and 2.

Reactome pathway	Matched IDs	*p* value	FDR
**A. Community 0**			
Antigen Presentation: Folding, assembly and peptide loading of class I MHC	35	1.11E−16	3.77E−15
Endosomal/Vacuolar pathway	35	1.11E−16	3.77E−15
Class I MHC mediated antigen processing and presentation	35	1.11E−16	3.77E−15
ER-Phagosome pathway	35	1.11E−16	3.77E−15
Antigen processing-Cross presentation	35	1.11E−16	3.77E−15
Immunoregulatory interactions between a Lymphoid and a non-Lymphoid cell	36	1.11E−16	3.77E−15
Interferon gamma signaling	36	1.11E−16	3.77E−15
Interferon alpha/beta signaling	36	1.11E−16	3.77E−15
SARS-CoV-2 activates/modulates innate and adaptive immune responses	35	1.11E−16	3.77E−15
Interferon Signaling	36	1.11E−16	3.77E−15
SARS-CoV-2-host interactions	35	1.11E−16	3.77E−15
SARS-CoV-2 Infection	35	1.11E−16	3.77E−15
SARS-CoV Infections	35	1.11E−16	3.77E−15
Cytokine Signaling in Immune system	39	1.54E−11	4.94E−10
Adaptive Immune System	37	2.03E−11	6.10E−10
Infectious disease	35	1.63E−05	4.56E−4
**B. Community 2**			
TRKA activation by NGF	2	1.49E−4	0.0400
DDX58/IFIH1-mediated induction of interferon-alpha/beta	4	4.98E−4	0.0400
Activation of TRKA receptors	2	5.88E−4	0.0400
Ca2+ activated K+ channels	2	5.88E−4	0.0400

## Discussion

In this manuscript, we applied spectral methods to analyze multiomics individual profiles from public data for 69 individuals. Our goals were to take an individual-focused approach and: (i) detect molecular-level deviations from each individual’s own baseline in response to dynamic changes in their physiological states, and (ii) build on the individual results to compare across subjects in a bottom-up approach and identify common molecular signatures. We generated periodograms for individual subject omics time series categorization, constructed within-person omics networks and detected personal-level immune changes corresponding to the individual’s physiological state changes. We identified similar individual-level responses to immune perturbation across multiple subjects. We then used the periodograms across subjects to identify network clusters of individuals with similarities across their common omics temporal patterns. The multi-individuals’ similarity network revealed different communities within which the molecular behavior was linked to phenotypic differences, including body mass index and insulin resistance, with the immune response dominating differences attributed to diabetic status, Table 3.

Our results are consistent with and extend previous research that has reported several cytokines with important roles in type 2 diabetes development, including cytokines from our findings across individuals in Table 2, with some examples highlighted below. Elevated concentrations of IP10 (CXCL10) have been reported in type 2 diabetes, and are associated with higher diabetes risk^29,30^. The IL13 pathway is a potential therapeutic target for glycemic control in type 2 diabetes^31^. IL27 has been implicated in insulin resistance in genome wide association studies^32^. Wang et al. had reported the pathogenic role of IL27, using diabetic NOD mice to investigate T-cell mediated autoimmune diabetes^33^, but recently the role of IL27-IL27R$\alpha$ in promoting adipocyte thermogenesis has been investigated in the context of treating insulin resistance^34^. Decreased plasma IL22 level was found to be a potential trigger of impaired fasting glucose and type 2 diabetes, in a retrospective study of Han Chinese subjects^35^. PDGFBB is reported as associated with type 2 diabetes mellitus and complications^36,37^. TGFA^38^ and TGFB^39^ have shown a pathologic contribution in diabetic kidney disease. TGFB is also reported associated with type 2 diabetic nephropathy^40^. ALKP has been investigated as an independent predictor for diabetes incidence^41,42^. MCP1 has been found significantly increased in patients with type 2 diabetes^43^. Furthermore, through rat studies GCSF has been reported as a potential novel therapeutic drug in early diabetic nephropathy patients^44^. The involvement of MIG (CXCL9) in the progression of type 2 diabetes nephropathy has been reported^45^. CD40-CD40L has been associated with type 2 diabetes mellitus^46^, VEGF is involved in the pathogenesis of diabetic complications^47^, c-Kit and its ligand, stem cell factor (SCF) have been reported as a potential novel target for treating diabetes^48^. Finally, chemotactic cytokines, including eosinophil chemotactic factors (ECFs), have been shown to be related to type 2 diabetes mellitus^49^. In our results, the cytokines above had high occurrence rates with statistically significant trends across the diabetic and prediabetic individuals. The findings suggest that our approach has potential application in clinical trials to identify disease biomakers and treatment targets.

In our analysis we used unsupervised methods to classify time-resolved trends in each subject, as well as to construct the network and identify the communities that showed differences in different diabetes-relevant measures (BMI, SSPG, DI and isrMax), Fig. 4 and Table 3. As the analysis did not depend on prior knowledge of the prediabetes/diabetes status of the subject, and given that the prediabetes-diabetes distinction is a rather continuous spectrum this suggests that potential pathophysiological signals are playing a part, and merits further future investigation.

Our study has limitations: In the current study we focused on immune changes and perturbations as these were available in the source data. As there are multiple other pathogenic factors that contribute to T2D, the data do not offer a complete picture and will need to be supplemented with additional followup studies. The data used in this investigation contained fine-grained omics data, and comparatively crude phenotypic data. Future investigations can address this at the outset of the experimental design prior to data collection. Furthermore, we have focused on immune markers, as these were the perturbation data available. To comprehensively study T2D more kinds of perturbations from baseline need to be investigated, including other disorders. Furthermore, the transcriptomic data used were generated from bulk RNA-sequencing, and hence do not allow for cell-type specific analyses, which are important is evaluating T2D. We expect that cell-type-specific studies with longitudinal data will become more prevalent with the recent focus on single-cell RNA-sequencing approaches. In terms of the data, there is uneven sampling (inherent in any real-world/subject based study), which we have addressed with our approach, as well as having different lengths of time series across individuals. Also, the RNA-seq data modality dominates in terms of number of omics. Our approach is robust as it analyzes single omics signals when assigning autocorrelation classes, irrespective of modality and hence is not affected by the unevenness between modality sizes. In the dendrogram constructions of determining groups and subgroups, it is possible that the trends are dominated by the large RNA-seq data, and potentially overfitting to a particular trend (i.e. missing subtle trend variation). Still, in this unsupervised clustering approach any distinct singleton omics patterns will be displayed.

Furthermore, environmental measures are not included and the low number of participants does not allow for a nuanced analysis of heterogeneity across subjects. For example, different subjects received different treatments which are analyzed in bulk. In terms of broad applicability of data collection and analysis, the data are obtained using invasive approaches (at least for blood components), which should improve with non-invasive transcriptomic mapping, such as using saliva. While our analysis included microbiome data (nares and gut), their association with transcriptome results, the cytokines and other measurements noted in Table 2, and their mechanistic role still requires further model-based experimentation. Finally, time series analyses with multiomics are an evolving area of research. We currently do not have T2D datasets that can be used for validation of findings. We anticipate that more studies will be generated that will also provide the necessary data to enable validation beyond the discovery approach in this investigation. Such data will also allow us to evaluate different methodologies, as there are many approaches to time-series analysis that we intend to continue investigating. In this investigation we have used the spectral methods as a first approach to detect data patterns in an unsupervised analysis. The spectral methods aid in addressing missing data/uneven sampling while maintaining the statistical properties of the data, and provide a streamlined pattern recognition based on frequency/autocorrelation behavior which is often used in time series analyses. Future work can also employ different approaches, especially for longer time series and with higher sampling rates to aid in the development of differential equation models of departures from signal baselines in response to perturbations, that provide more mechanistic interpretations of T2D dynamics.

In this manuscript we performed an individual-focused analysis of a prediabetes study cohort^4,14^, which revealed insights from this longitudinal dataset and can lead to actionable health discoveries, providing relevant information for precision health monitoring. Our approach is the first, to our knowledge, with a temporal bottom-up/individual focus. Starting from individual microscopic measurements (molecular level omics), intra-subject immune responses can be characterized. Then, building on the individual responses, a macroscopic inter-subject temporal clustering of subjects based on temporal similarity provides information on how immune responses can be related to diabetic states, consistent with and supplementing the original work.

In summary, our findings utilize personal temporal omics to identify collective responses across individuals associated with macroscopic characteristics, and provide an approach that can potentially help predict disease responses and outcomes towards clinical implementations. The approach is non-disease specific, and extensible: any time signal measurement can be incorporated and any number of individuals can be compared spanning periods of individualized wellness and departures therefrom. Additionally, real-world application limitations of missing data and uneven sampling can be addressed. Beyond identifying trends within an individual, expanding to larger cohorts can eventually provide individual temporal signatures of specific disease onset. Such temporal disease signatures can be used to train models for monitoring departures from a healthy baseline, towards prevention or minimally timely treatments. Coupling multi-timepoint monitoring with non-invasive sampling (e.g. saliva^12^) can help eventually provide affordable population-level precision health.

## Data Availability

The original data analyzed in this investigation were made publicly available by Zhou et al.^14^ and Schussler-Fiorenza Rose et al.^4^ as described therein, on https://med.stanford.edu/ipop.html. All data files used in this investigation, including original code and results files have been released on Github (https://github.com/gmiaslab/TemporalMultiomicsDiabetes), and also deposited on Zenodo (10.5281/zenodo.6751960), and are referred to as Online Data Files (ODFs) in the manuscript.
